# Is virtual reality beneficial for dual-task gait training in patients with Parkinson's disease? A systematic review

**DOI:** 10.1590/1980-57642018dn13-030002

**Published:** 2019

**Authors:** Fernanda Freitag, Sonia Maria Dozzi Brucki, Alessandra Ferreira Barbosa, Janini Chen, Carolina de Oliveira Souza, Débora Francato Valente, Hsin Fen Chien, Cynthia Bedeschi, Mariana Callil Voos

**Affiliations:** 1 FMUSP Department of Neurology Postgraduate Program in Neurology São PauloSP Brazil Postgraduate Program in Neurology, Department of Neurology, FMUSP, São Paulo, SP, Brazil.; 2 Rehabilitation in Movement Disorders (REMOVE) Research Group São PauloSP Brazil Rehabilitation in Movement Disorders (REMOVE) Research Group,São Paulo, SP, Brazil.; 3 FMUSP Physiotherapy, Speech Therapy and Occupational Therapy Postgraduate Program in Rehabilitation Sciences São PauloSP Brazil Postgraduate Program in Rehabilitation Sciences. Physiotherapy, Speech Therapy and Occupational Therapy, FMUSP, São Paulo, SP, Brazil.; 4 USP Institute of Psychology Postgraduate program in Neuroscience and Behavior São PauloSP Brazil Postgraduate program in Neuroscience and Behavior, Institute of Psychology, USP, São Paulo, SP, Brazil.

**Keywords:** Parkinson's disease, gait, cognition, virtual reality, videogames, doença de Parkinson, marcha, cognição, realidade virtual, videogames

## Abstract

This systematic review examined the evidence about the effects of virtual reality (VR) on dual-task gait training in Parkinson's disease (PD).

**Methods:**

this study (PROSPERO registration CRD42019114736) aimed to answer the question: “Is VR beneficial for dual-task gait training in patients with PD?” We searched for studies from 2008 to 2018 on Medline/PubMed and Web of Science/Web of knowledge databases. The keywords were Parkinson AND gait training AND virtual reality OR Parkinson AND gait training AND game. A total of 55 articles were retrieved, of which 11 systematic reviews, 11 opinions, letters to the editor, posters or conferences abstracts and 17 studies not evaluating the effects of VR gait training were excluded. Three further studies addressing VR dual-task gait training in PD (found in references of studies selected) were also included. Therefore, 19 studies were included and analysed.

**Results:**

all studies reported gait improvement after VR training. Many clinical scales were used, hampering comparison of the effects of each protocol.

**Conclusion:**

VR dual-task gait training should be part of rehabilitation protocols for PD. The studies showed that VR training was effective, although specific guidelines have not yet been established.

## RESUMO

Essa revisão sistemática examinou as evidências sobre os efeitos da realidade virtual (RV) no treinamento de marcha com tarefa-dupla na doença de Parkinson (DP).

**Métodos:**

esse estudo (registro PROSPERO: CRD42019114736) visou responder à questão: “A RV é benéfica no treinamento de marcha com tarefa-dupla em pacientes com DP?” A busca, de 2008 a 2018, foi feita nos bancos de dados Medline/PubMed e Web of Science/Web of Knowledge. Os descritores foram Parkinson, treinamento de marcha, realidade virtual e jogos eletrônicos. Foram encontrados 55 artigos e eliminadas 11 revisões sistemáticas, 11 cartas ao editor, resumos de pôsteres ou conferências e 17 estudos que não avaliaram os efeitos do treinamento da marcha em RV. Três outros estudos (encontrados nas referências dos estudos selecionados) foram incluídos porque também abordaram o treinamento de marcha com dupla tarefa de RV em DP. Portanto, 19 estudos foram incluídos e analisados.

**Resultados:**

todos os estudos relataram melhora da marcha após o treinamento com RV. Muitas escalas clínicas foram usadas e foi difícil comparar os efeitos de cada protocolo.

**Conclusão:**

RV deve fazer parte dos protocolos de reabilitação em DP. O treinamento com RV é eficaz, embora recomendações específicas ainda não tenham sido estabelecidas.

Parkinson's disease (PD) is the second most common neurodegenerative disease, characterized by motor and cognitive symptoms. Executive dysfunction can be present from the early stages of PD. These deficits increase falls risk and reduce functional independence, especially in dual-task performance. Executive function has been defined as a group of abilities involved in solving problems, reaching goals and meeting environmental demands,^1^^,^^2^ such as cognitive flexibility and decision-making.^3^ More than 50% of patients with PD have frontal lobe dysfunction, which compromises attention, executive function, spatial perception and implicit/episodic memory.^4^

Patients with PD have a poorer standing balance than healthy individuals.^5^^,^^6^ While performing a secondary task associated with ambulation, patients with PD have lower gait speed, shorter step length and freezing of gait episodes. Postural control deficits result in decreased mobility and reduced functional independence,^6^ e.g. during the sit-to-stand-to-sit sequence, especially when under the dual-task condition. Therefore, patients with PD must deal with the disruption of their motor and cognitive performance when tackling the cognitive-motor demands of their tasks of daily living.^6^^,^^7^

Many studies have shown the importance of visual, auditory, verbal and mnemonic cues (e.g. mental practice). Although cues help attention engagement during balance and gait tasks^2^^,^^4^^,^^8^ they may compete with other cognitive components required in daily life dual- or multiple-tasks. For example, while crossing a street, the patient may evoke visual or auditory cues to maintain step length or cadence. However, this strategy may compete with the intrinsic visual and auditory stimuli of traffic lights, pedestrians and vehicles.

Dual-task training improves cognitive-motor performance and directs attentional focus on specific outcomes. This is important because of the similarity with everyday tasks, when people focus on outcomes rather than on maintaining balance.^9^ Virtual reality (VR) provides dual-task training and requires information processing, attentional shifting, sensory integration, motor planning, while VR may also provide feedback to enhance motor learning.^9^^,^^10^

Postural control requires the integration of visual, somatosensory and vestibular systems. Executive function contributes in adapting inputs to meet environmental demands. In older adults and patients with PD, the reduced speed of sensory processing, motor planning and muscle activation results in increased attentional demands to maintain stability.^11^ VR training may optimise all these components and, therefore, motor learning. It may also be safer and more motivating than conventional approaches.^11^^-^13 Thus, VR training may improve adherence by offering personalised and fun exercises, with progressive cognitive overload.^14^

VR provides complex environments for balance and gait training of patients with PD. Improvements in step and stride length, gait velocity, functional independence, quality of life, and cognitive function have been reported after VR interventions with PD patients.^14^^,^^15^. Therefore, VR can play an important role in motor control and learning in PD.^16^ Several VR dual-task gait training protocols have been proposed for patients with PD, but the effects are not clear. This systematic review aimed to examine and analyse the evidence on VR dual-task gait training in PD.

## METHODS

This systematic review followed the Preferred Reporting Items for Systematic Reviews and Meta-Analyses (PRISMA) checklist. The study was registered on the International Prospective Register of Systematic Reviews (PROSPERO) under registration number CRD42019114736. Two researchers conducted the data search independently and blindly. Another three researchers conducted the data review and all researchers discussed all cases of doubt. The study aimed to answer the question: “Is VR beneficial for dual-task gait training in patients with PD?”

The inclusion criteria were studies available on Medline/ Pubmed and Web of Science/ Web of Knowledge databases, which addressed VR dual-task gait training. The exclusion criteria were: studies that focused on drugs or surgery instead of dual-task training, literature reviews, abstracts and letters to the editor.

We searched studies published from 2008 to 2018 on Medline/ Pubmed and Web of Science/ Web of Knowledge databases. The keywords were *Parkinson* AND *gait training AND virtual reality OR Parkinson AND gait training AND game.*

**Figure 1:**
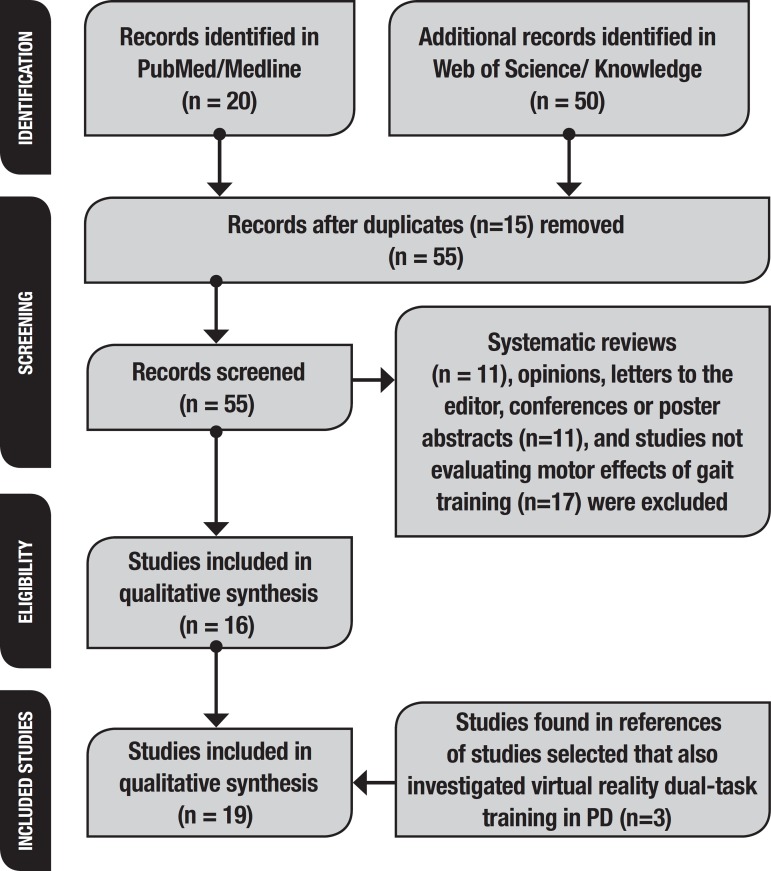
Studies included in systematic review, based on PRISMA criteria (2009): identification, screening, eligibility and studies included in review (n=19).

The search was performed on August 26^th^, 2018. A total of 55 articles were retrieved , of which 11 systematic reviews, 17 studies not specifically evaluating the effects of VR dual-task gait training and 11 opinions, letters to the editor or conference or poster abstracts were subsequently excluded. The references of these sixteen studies were also analysed. Three further studies focusing on VR dual-task gait training in PD were also included. Therefore, 19 studies were included in the qualitative synthesis (Figure 1). Figure 1 shows all the steps of the systematic review and the reasons for study exclusions.

The studies were also scored with the Physiotherapy Evidence Database (PEDro). This database evaluates the quality of each study and the validity of their conclusions based on the Delphi list (Verhagen et al., 1998).^17^ This scale has 11 domains, which evaluate: 1. Eligibility criteria; 2. Random distribution of subjects in each group; 3. Secret allocation of subjects; 4. Similar groups regarding the most important prognosis; 5. Blind participation of subjects; 6. Blind participation of therapists; 7. Blind examiners; 8. At least one key result obtained in more than 85% of subjects; 9. Subjects received treatment or control condition; 10. Intergroup statistical comparisons have been performed for at least one key outcome; 11. Presence of precision and variability measures.

## RESULTS

The 19 studies selected are summarized in Table 1. Several clinical scales were used, but the most frequent were Parkinson's Disease Questionnaire-39, Unified Parkinson Disease Rating Scale-III, Dynamic Gait Index and the 10-Minute Walk Test. Most studies described gait improvement after virtual reality training. The six-minute walk test, the Montreal Canadian Cognitive Assessment, the timed up-and-go test and the Berg Balance Scale were also used in evaluation protocols, but less frequently.

**Table 1:** Synthesis of the 19 studies included in the systematic review.

Authors	Participants	Method	Main findings
Espay et al., 2010^18^ At-home training with closed-loop augmented reality cueing device for improving gait in patients with PD.	• 13 PD patients • Age: 73.3±11.7 yrs • PD duration: 12.1±4.2 yrs • H&Y: not available	The efficacy of an accelerometer-driven, wearable, visual-auditory cueing device at baseline and after 2 weeks of twice daily (30 min) at-home use was examined. Gait velocity, stride length, and cadence were measured using a validated electronic gait-analysis system. FOG-Q was used for clinical assessment.	After training, patients improved velocity and stride length with the device. The effects were maintained even after the device was removed. Devices with closed-loop sensory feedback appear to be effective and desirable interventions to improve gait in PD patients. An overall improvement in gait was detected by the FOG-Q.
Mirelman et al., 2011a^19^ VR for gait training: can it induce motor learning to enhance complex walking and reduce fall risk in patients with PD?	• 20 PD patients • Age: 67.1±6.5 yrs • PD duration: 9.8±5.6 yrs • H&Y: 2-3	Eighteen sessions (three per week) of progressive intensive treadmill training with virtual obstacles. Outcome measures included gait with single and dual-tasks and while negotiating obstacles. Functional performance was assessed by UPDRS-III, four square step test, PDQ-39, MoCA and TMT.	Gait speed improved during single-tasks and while negotiating obstacles. Dual-task gait variability, TMT times (parts A and B), scores on UPDRS-III and four square step test performance improved). Functional gains were also observed one month later (retention effects). Quality of life increased after training.
Esculier et al., 2012^20^ Home-Based balance training program using Wii Fit^TM^ with balance board for PD: A pilot study.	• 9 healthy subjects (age: 63.5±12.0 yrs) • 11 PD patients (age: 61.9±11.0 yrs, PD duration: 8.5±3.6).	Approximately 40 min, 3 days/week for 6 weeks (18 total training sessions) with pre-determined number of repetitions of game trials. Each training day included 30 min with the Wii Fit^TM^ using the balance board and 10 min with the Wii Sports^TM^ game (golf or bowling).	Balance, global mobility and functional abilities improved in PD patients (measured by UPDRS, Activities-specific Balance and Confidence Scale, TUG, Sit-to-Stand Test, Performance-Oriented Mobility Assessment, Community Balance and Mobility scale, 10MWT. Healthy subjects also improved, but to a lesser extent.
Mhatre et al., 2013^21^ Wii Fit balance board playing improves balance and gait in PD.	• 10 subjects with PD (4 men) • Age: 67.1 yrs (44-91 yrs), SD was not informed. • PD duration: 6.7 yrs (1-14 yrs), SD was not informed. • H&Y: 2.5-3.	Nintendo Wii Fit^TM^ and balance board system effects on balance and gait were evaluated with BBS, DGI and Romberg. Training occurred 3 times a week for 8 weeks for 30 min. Wii balance board^TM^ measured postural sway. Balance confidence was rated by the Activities-specific Balance Confidence scale and depression by GDS.	The Wii Fit^TM^ balance board training improved balance and gait. No significant changes were seen in mood or confidence. BBS increased significantly. DGI improved, as did postural sway (decreased 31% variance in stance with eyes open), but these differences were non-significant. Romberg with eyes closed increased significantly.
Pompeu et al., 2014^22^ Feasibility, safety and outcomes of playing Kinect Adventures! for people with Parkinson's disease: a pilot study.	• 7 PD patients (6 males) • Age: 72.0±9.0 yrs • PD duration: not informed • H&Y 2-3.	Kinect Adventures! training consisted of fourteen 60-min sessions, 3 times a week. Feasibility and safety outcomes included game performance and adverse events. Clinical outcomes were 6MWT, Balance Evaluation System Test, DGI and PDQ-39.	The scores of all games, 6MWT, Balance Evaluation System Test, DGI and PDQ-39 improved. Kinect-based training was safe and feasible. Kinect Adventures! improved balance, gait, cardiopulmonary aptitude and quality of life.
Esculier et al., 2014^23^ Corticomotor excitability in PD during observation, imagery and imitation of action: effects of rehabilitation using Wii Fit and comparison to healthy controls.	• Eight subjects with moderate PD (age: 63.5±12 yrs, PD duration: 8.5±3,6 yrs) and eight controls (age: 61.9±12 yrs).	This study compared lower limb corticomotor activation during action observation, motor imagery, and imitation, and evaluated the effects of a 6-week training program using Wii Fit^TM^ on corticomotor excitability. Transcranial magnetic stimulation assessed motor evoked potentials in quadriceps femoris and soleus muscles after six weeks of training using Wii Fit^TM^ (mini-squat).	PD group showed less evoked potentials in quadriceps in observation, motor imagery and imitation compared to controls (baseline). In soleus, evoked potentials were reduced in PD group in imitation. PD group increased quadriceps evoked potentials in observation, in contrast to controls, after training. Both groups reduced motor evoked potentials in quadriceps and soleus in imitation. PD group improved on TUG and Sit-to-Stand.
Killane, et al., 2015^24^ Dual motor-cognitive VR training impacts dual-task performance in freezing of gait.	• FOGPD (n=13): 64.2±2.4 yrs; H&Y: 2.6±0.1; • Non-FOGPD (n=7): 64.0±1.6 yrs; H&Y: 2.3±0.1; • PD duration: not available	A VR dual-task intervention combined motor and cognitive tasks to improve dual-task performance. Patients were assessed with MoCA, FAB, UPDRS III and FOG.	Dual-task performance improved post-intervention in patients with FOG, who also showed a decrease in the number of FOG episodes.
Liao et al., 2015^25^ VR-based Wii fit training in improving muscle strength, sensory integration ability and walking abilities in patients with PD.	• 36 PD patients (H&Y 1-3) • Control 64.6±8.6 yrs; PD duration: 6.4±3.0 yrs • Traditional exercise: 65.1±6.7 yrs, PD duration: 6.9±2.8 yrs; • VR-based Wii Fit^TM^ exercise: 67.3±7.1 yrs; PD duration: 7.9±2.7 yrs	The effects of VR-based training in improving muscle strength, sensory integration ability, and walking abilities in PD patients after training and after 1-month follow-up were evaluated in this study. UPDRS, MMSE, GAITRite, Functional Gait Assessment, Body Mass Index were used for assessment.	Wii fit^TM^ improved stride length and velocity compared with the controls after training and at follow-up. No differences between Wii fit^TM^ and traditional exercises were found (both groups improved velocity and sensory integration compared to controls after training and at follow-up). VR-based Wii fit^TM^ exercise and traditional exercises improved gait, sensory integration, and muscle strength.
Liao et al., 2015^26^ VR-based training to improve obstacle-crossing performance and dynamic balance in patients with PD.	• 36 PD patients (H&Y 1-3). • Control 64.6±8.6 yrs; PD duration: 6.4±3.0 yrs • Traditional exercise: 65.1±6.7 yrs, PD duration: 6.9±2.8 yrs; • VR-based Wii Fit^TM^ exercise: 67.3±7.1 yrs; PD duration: 7.9±2.7 yrs	The effects of virtual reality-based exercise on obstacle crossing performance and dynamic balance were evaluated in PD patients. PDQ-39, FES-I and TUG were used for assessment.	The VR Wii^TM^ group showed greater improvement in obstacle crossing velocity, crossing stride length, dynamic balance, sensory organization test, TUG, FES-I, and PDQ-39 than the control group. VR Wii^TM^ training also resulted in greater improvement in movement velocity of limits-of-stability test than traditional exercises.
Palacios-Navarro et al., 2015^27^ A Kinect-based system for lower limb rehabilitation in PD: a pilot study.	• Seven PD patients • Age: 66.8±3.5 yrs • PD duration: not available • H&Y: not available.	The effects of VR-based exercise on obstacle crossing performance and dynamic balance were evaluated in PD patients by the MMSE and the 10MWT.	Patients improved on the 10 MWT. Feedback from participants supports the use of VR not only in rehabilitation centers but also at home.
Ginis et al., 2016^28^ Feasibility and effects of home-based smartphone-delivered automated feedback training for gait in people with PD: a pilot randomized controlled trial.	• Smartphone application group (CuPiD-systems, n=22): 67.3±8.1 yrs. PD duration: 10.6±5.3 yrs. H&Y: 2-3. • Control group (n=18): 66.1±8.0 yrs. PD duration: 11.6±7.6 yrs. H&Y: 2-3. Distribution was randomized.	The smartphone provided feedback on gait performance. Feasibility and effectiveness were investigated in home environment. Gait training lasted for 30 min, 3 times a week, for 6 weeks. Gait, balance, endurance and quality of life were assessed with MiniBESTest and SF-36 after training and at 1 month-follow-up.	Both groups improved in single- and dual-task gait speed at post-test and follow-up. The smartphone group improved significantly more on balance at post-test and maintained quality of life at follow-up. whereas the control group deteriorated. The smartphone system was well-tolerated, and participants found the tool user-friendly.
Yang et al., 2016^29^ Home-based virtual reality balance training and conventional balance training in PD: a randomized controlled trial.	• VR (n=11): 72.5±8.4 yrs, PD duration: 9.4±3.6 yrs, H&Y: 3. • Control (n=12): 75.4±6.3 yrs, PD duration: 8.3±4.1 yrs, H&Y: 3.	The effects of VR balance training were evaluated in patients with PD to investigate whether VR balance training would be superior to conventional balance training. Patients were evaluated with BBS, DGI, TUG, PDQ-39, UPDRS III.	No differences between VR training and conventional training were found.
Strouwen et al., 2017^30^ Training dual tasks together or apart in PD: results from the duality trial.	• 121 PD patients • group 1: age: 66.1±9.3 yrs; PD duration: 8.9±6.3 • group 2: age: 65.8±9.2; PD duration: 8.4±5.3 • H&Y: 2-3 • Distribution was randomized	Two training programs (six weeks) were compared: consecutive (gait and cognitive tasks trained separately) and integrated (gait and cognitive tasks trained simultaneously). Two baseline tests were performed as a six-week control period before training (MoCA and FAB). Post-tests were performed after training and at 12-week follow-up. Falls risk was determined by weekly calls for 24 weeks.	Both protocols had similar effects on dual-task gait. Improvements in dual-task gait velocity were found and were retained at 12-week follow-up. No significant change in fall risk occurred in either group.
Gandolfi et al., 2017^31^ VR telerehabilitation for postural instability in PD: a multicenter, single-blind, randomized, controlled trial.	• 76 PD patients • Nintendo Wii Fit^TM^ (n=36): 67.5±7.2 yrs, PD duration: 6.2±3.8 yrs, H&Y: 2.5. • sensory integration balance training (n=34): 69.8±9.4 yrs, PD duration: 7.5±3.9 yrs, H&Y: 2.5-3.0.	Postural stability after in home VR-based balance training with the Nintendo Wii Fit^TM^ and after in-clinic sensory integration balance training were compared. Balance confidence, mobility-related function, quality of life, falls, UPDRS, GDS and the costs of the rehabilitation programs were evaluated.	BBS scores improvement was significant after 7 weeks (completion of training programs) and at follow-up evaluation. Both groups showed improvement on the 10MWT, DGI, and PDQ-39. Nintendo Wii Fit^TM^ holds promise and potential to enrich rehabilitation care at home in patients with PD.
Ferraz et al., 2018^32^ The effects of functional training, bicycle exercise, and exergaming on walking capacity of elderly patients with PD: a pilot randomized controlled single-blinded trial.	• Group 1 (n=22, 16 men): 71.0 ±5.0 yrs, H&Y: 2.5-3.0. PD duration: 4.0±3.0 yrs • Group 2 (n=20, 11 men): 67.0 ±4.0 yrs, H&Y:2.0-3.0. PD duration: 6.0±4.0 yrs • Group 3 (n=20,10 men): 67.0 ±4.0 yrs, H&Y: 2.0-2.5. PD duration: 4.0±3.0 yrs	This study compared the effects of 3 treatment modalities (functional training, bicycle exercise, and exergaming) on gait of PD patients. Patients were evaluated with UPDRS, MMSE, 6MWT, 10MWT, PDQ-39, body mass index, world health organization disability assessment schedule, sitting-rising test and GDS.	All groups showed significant improvements on 6MWT and SRT. Group 3 improved gait speed on 10MWT. Groups 1 and 3 improved quality of life. Functional training, bicycle exercise, and Kinect Adventures exergames were safe and improved the walking capacity of patients with PD.
Dantas et al., 2018^33^ Training healthy persons and individuals with PD to use Xbox Kinect games: a preliminary study.	• 19 adults (8 with PD and 11 healthy adults). • PD Group: 65.6±11.8 yrs. H&Y: 1-3, PD duration: not mentioned • Healthy Adults Group: 70.0±7.7 yrs	This study investigated the effects of motor and cognitive demands of six Kinect for Xbox 360^TM^ games (Target Kick, Stack 'em Up, Wall Breaker, Super Saver, Paddle Panic and Bump Bash) on the learning of PD patients, compared to healthy individuals. MMSE were used in the assessment.	Both groups improved their performances in terms of the scores obtained in each session compared to the first session on Target Kick, Stack 'em Up, Wall Breaker and Super Saver. Motor and cognitive abilities improved with the use of VR. Some of the games and devices can influence the learning process, even in healthy adults.
Melo et al., 2018^34^ Effect of virtual reality training on walking distance and physical fitness in individuals with PD.	• 37 PD patients. • Control group (n=12): 65.6±13.0 yrs, H&Y: 2.08±0.9 • Treadmill group (n=13): 61.0±10.7 yrs, H&Y: 1.53±0.66 • VR group (n=12): 60.3±9.3 yrs, H&Y1.41±0.51 • PD duration: not mentioned	This study evaluated the effects of VR gait training on walking distance and physical fitness. The control group was submitted to conventional training, the treadmill group was submitted to gait training on a treadmill and the VR group was submitted to gait training using the Xbox^TM^. Patients were evaluated with 6MWT, UPDRS III and PDQ-39.	Heart rate increased during the intervention in VR and treadmill groups. HR variation was more intense in VR group after the ﬁrst training session and after training. Gains were not maintained at 30 days after training. Treadmill training was more effective at maintaining physical ﬁtness than VR activities. VR was as effective as treadmill training for improving walking distance and temporal gait variables.
Alves et al., 2018^35^ Nintendo Wii versus Xbox Kinect for assisting people with PD.	• 27 patients (25 men): 61±10.7 yrs. • Nintendo Wii^TM^ group (n=9): 58.9±11.2 yrs, H&Y: 1.9± 0.9. • Xbox Kinect group (n=9): 62.7±13.8 yrs, H&Y: 1.6± 0.7. • Control group (n=9): 61.7±10.7 yrs, H&Y: 1.8± 0.8.	This study compared the effects of 10 VR sessions with Nintendo Wii^TM^ and Xbox Kinect on motor and cognitive performance, anxiety levels, and perceived quality of life changes in patients with PD. Patients were evaluated with MMSE, GDS, WHOQOL-OLD, Beck Anxiety Inventory, VFT, Digit Span (forward and backward), TUG, 10MWT, 30-Second Walk Test.	Improvements in gait performance after Nintendo Wii^TM^ training were evident on the 30-Second Walk Test on both single and dual tasks (increase in distance covered and decrease in number of steps taken). Anxiety scores and Digit Span Backward scores decreased after Nintendo Wii^TM^ training. The Xbox Kinect^TM^ did not facilitate these improvements. Greater benefits were observed in the simpler and less distracting interface of the Nintendo Wii^TM^.
Song et al., 2018^36^ Home-based step training using videogame technology in people with Parkinson's disease: a single-blinded randomised controlled trial.	• Intervention group: n=31, 68.0±7.0, PD duration: 7±4 yrs and control group: n=29, 65.0±7.0, PD duration: 9±6 yrs	This study aimed to determine whether 12-week home-based exergame step training could improve stepping performance, gait and neuropsychological measures associated with falls in PD. Choice stepping reaction time test, Functional Gait Assessment and neuropsychological functions, number of falls over six months and self-reported mobility and balance were evaluated.	Post-intervention, there were no differences between the intervention and control groups except for the TUG (the difference favoured the control group). Intervention participants reported mobility improvement, whereas control participants reported mobility deterioration. Interaction effects between intervention and disease severity on physical function measures were observed with seemingly positive effects for the low-severity group and potentially negative effects for the high-severity group.

BBS: Berg Balance Scale; DGI: Dynamic Gait Index; FAB: Frontal Assessment Battery; FES-I: Fall Efficacy Scale; FOG-Q: Freezing of gait questionnaire; GAITRite: Assessment of level of walking performance; GDS: Geriatric Depression Scale; H&Y: Hoehn & Yahr Staging Scale; MiniBESTest: Mini Balance Evaluation Systems Test; MMSE: Mini-Mental State Examination; min: minutes; MoCA: Montreal Cognitive Assessment; PD: Parkinson's disease; PDQ-39: Parkinson's Disease Questionnaire-39; SD: standard deviation; SRT: serial reaction time; SF-36: Short Form Health Survey; TMT: Trail-Making Test; TUG: Timed Get Up and Go Test; UPDRS-III: Unified Parkinson's Disease Rating Scale - Part III; VFT: Verbal Fluency Test; VR: virtual reality; WAIS-III: Wechsler Adult Intelligence Scale; WHOQOL-OLD: World Health Organization Quality of Life for Older Persons; yrs: years; 6MWT: Six-Minute Walk Test; 10 MWT: Ten-Meter Walk Test.

Table 2 shows the score on each domain of the PEDro database. The scores on PEDro ranged from 4 to 11, but 11 studies were scored as 8 or higher. Therefore, although protocols were variable, many studies showed high quality of evidence that supported VR dual-task gait training.

**Table 2:** PEDro database classification.

Authors	1	2	3	4	5	6	7	8	9	10	11	Total
Espay et al., 2010^18^	Yes	Yes	No	Yes	No	No	No	Yes	Yes	Yes	Yes	7
Mirelman et al., 2011^19^	Yes	No	No	No	No	No	No	Yes	Yes	No	Yes	4
Esculier et al., 2012^20^	Yes	No	No	No	No	No	No	Yes	Yes	No	Yes	4
Mhatre et al., 2013^21^	Yes	Yes	No	Yes	No	No	No	Yes	Yes	Yes	Yes	7
Esculier et al., 2014^22^	Yes	No	No	Yes	No	No	No	Yes	Yes	No	Yes	5
Pompeu et al., 2014^23^	Yes	No	No	Yes	Yes	Yes	Yes	Yes	Yes	No	Yes	8
Killane, et al., 2015^24^	Yes	No	No	Yes	No	No	No	Yes	Yes	Yes	Yes	6
Liao et al., 2015^a^^25^	Yes	Yes	Yes	Yes	No	Yes	Yes	Yes	Yes	Yes	Yes	10
Liao et al., 2015^b^^26^	Yes	Yes	Yes	Yes	No	Yes	Yes	Yes	Yes	Yes	Yes	10
Palacios-Navarro et al., 2015^27^	Yes	No	No	Yes	No	No	No	Yes	Yes	No	Yes	5
Ginis et al., 2016^28^	Yes	Yes	No	Yes	Yes	No	No	Yes	Yes	Yes	Yes	8
Strouwen et al., 2017^29^	Yes	No	Yes	Yes	Yes	Yes	Yes	No	Yes	Yes	Yes	9
Yang et al., 2016^30^	Yes	Yes	Yes	Yes	No	No	No	Yes	Yes	Yes	Yes	8
Gandolfi et al, 2017^31^	Yes	Yes	No	Yes	No	Yes	Yes	Yes	Yes	Yes	Yes	9
Ferraz et al., 2018^32^	Yes	Yes	Yes	Yes	Yes	No	No	Yes	Yes	Yes	Yes	9
Dantas et al., 2018^33^	Yes	No	No	No	No	No	No	Yes	Yes	Yes	Yes	5
Melo et al., 2018^34^	Yes	Yes	Yes	Yes	Yes	Yes	Yes	Yes	Yes	Yes	Yes	11
Alves et al., 2018^35^	Yes	Yes	Yes	Yes	No	No	No	Yes	Yes	Yes	Yes	8
Song et al., 2018^36^	Yes	Yes	Yes	Yes	Yes	Yes	Yes	Yes	Yes	Yes	Yes	11

1. Eligibility criteria; 2. Random distribution of subjects in each group; 3. Secret allocation of subjects; 4. Similar groups regarding the most important prognosis; 5. Blind participation of subjects; 6. Blind participation of therapists; 7. Blind examiners; 8. At least one key result obtained in more than 85% of subjects; 9. Subjects received treatment or control condition; 10. Intergroup statistical comparisons have been performed for at least one key outcome; 11. Presence of precision and variability measures.

## DISCUSSION

The present study investigated whether VR dual-task gait training would promote gait improvement in PD patients. Patients with PD experience loss of functional independence and quality of life and difficulties in activities of daily living. VR can increase motivation in rehabilitation programs, and improve gait, as observed in the nineteen studies from 2008 to 2018 included in the present review. Although the scores on the PEDro scale ranged from 4 to 11, 11 studies were scored as 8 or higher. Therefore, many studies showed high quality of evidence that supported VR dual-task gait training.

VR opens a wide range of possibilities of therapeutic approaches, and involves several types of stimuli (sensory, motor, cognitive, psychological). Thus, VR training provides several dual-task demands, characterizing one of the training premises. Therefore, VR may optimize gait training through posture and balance improvement and cognitive training.^20^^,^^23^^,^^37^ Most VR tasks are cognitive-motor, such as activities of daily living.^6^^,^^7^

Many studies included in the present review showed improvements in speed, step length and cadence of gait, measured by kinematic analysis and/ or clinical scales, such as the Dynamic Gait Index, Timed Up-and-Go, 6-minute walking test or 10-meter walking test (Espay et al., 2010;^18^ Mirelman et al., 2011;^19^ Esculier et al., 2012;^20^ Mhatre et al., 2013;^21^ Pompeu et al., 2014;^22^ Liao et al., 2015;^25^ Palacios-Navarro et al., 2015;^27^ Ginis et al., 2016;^28^ Yang et al., 2016;^29^ Strouwen et al., 2017;^30^ Gandolfi et al., 2017;^31^ Ferraz et al., 2018;^32^ de Melo et al., 2018;^34^ Alves et al., 2018^35^). The improvement in gait speed may be attributed to higher motivation, or to the higher intensity (higher number of repetitions and/ or longer periods of training) promoted by the VR dual-task training. These training quality characteristics may also explain the more efficient cardiovascular adjustments, e.g. heart rate (de Melo et al., 2018^34^).

Some authors reported improvement on static postural balance, as measured by the Berg Balance Scale, after VR dual-task gait training (Mhatre et al., 2013;^21^ Pompeu et al., 2014;^22^ Yang et al., 2015;^29^ Gandolfi et al., 2017^31^). These protocols involved weight shifting and functional reach tasks, highly recommended in PD rehabilitation because they improve static and dynamic balance. The association of static and dynamic balance and gait speed improvement may explain the less severe PD motor symptoms after training, measured by the UPDRS-III motor score, as reported by Mirelman et al., 2011;^19^ Killane et al., 2015;^24^ Liao et al., 2015;^25^ Yang et al., 2015;^29^ Gandolfi et al., 2017;^31^ Ferraz et al., 2018;^32^ de Melo et al., 2018.^34^

VR dual-task gait training involves not only motor, but also cognitive training. Executive function, evaluated by parts A and B of the Trail-Making Test, improved in patients with PD after the training (Mirelman et al., 2011^19^). Patients also reported improvement in PD symptoms (Sony et al., 2018)^36^ and in quality of life, assessed by the PDQ-39 (Pompeu et al., 2014;^22^ Liao et al., 2015;^26^ Yang et al., 2015;^29^ Ferraz et al., 2018;^32^ de Melo et al., 2018^34^).

Some authors suggest that VR tasks can improve motor learning in rehabilitation because they activate mirror neurons. When patients with PD imitate actions, mirror neurons are stimulated, allowing improvements in balance, global mobility and functional abilities. In addition, VR improves attention as patients focus on specific demands of games by recruiting cognitive, motor, oculomotor, cerebellar and limbic loops.^37^^,^^38^

Patients with PD have difficulty performing dual- or multiple-tasks.^5^^-^7 This difficulty occurs because patients must focus on specific and accurate motor patterns. Thus, the premotor cortex is activated to compensate for basal ganglia damage and deficiency in dopamine production.^38^^,^^39^ Therefore, in dual-tasks, cortical resources process the motor and cognitive components in parallel.^38^^-^40 Patients with PD employ their cognitive reserves to perform gait even in single-tasks, and performance is seriously impacted in dual-tasks.^41^

Dual-task training should be part of the rehabilitation process of PD patients who have difficulty performing cognitive-motor tasks.^42^^,^^43^ Besides, patients in the initial stages of PD should perform dual-task training to prevent or delay these deficits.^42^ In a recent study, Fernandes et al. (2017)^44^ showed that the anticipatory postural adjustments during gait initiation were impaired in patients with PD. The authors reported an activation failure of the tibialis anterior muscle in both single- and dual-task conditions. Therefore, exercises that involve repeated tibialis anterior activation, such as step climbing,^36^ are important and should be included in rehabilitation programs for patients with PD. Song et al. (2018) combined VR with a step climbing task.^36^ Although patients reported mobility improvement, the authors failed to find a significant effect of VR step climbing training on TUG performance. Based on the study by Fernandes et al. (2017), an electromyographic-based analysis may be more sensitive for detecting postural control improvement than TUG, particularly in early-stage PD patients.^44^

The studies reviewed in the present study showed that VR dual-task training is effective, although the specific guidelines of dual-task protocols have not yet been defined.^39^ VR optimizes the benefits of dual-task training, such as task automation and more efficient task-related network integration.^30^ Visuomotor training can help the reorganization and maintenance of the normal circuitry that connects the motor cortex with the basal ganglia via the thalamus or cerebellum.^43^ Visuomotor training can involve temporal or spatial stimuli, which regulate and facilitate repetitive movements by providing explicit targets. Visual cues have immediate effects on gait and many studies shown that effects were retained and associated with a higher quality of life in PD.^45^^-^47

The study by Esculier et al. (2014)^23^ showed that lower limb corticomotor excitability increased during the observation, imagery and imitation of actions. Transcranial magnetic stimulation assessed motor evoked potentials in quadriceps femoris and soleus muscles before and after six weeks of training. The authors compared the effects of rehabilitation using Wii Fit^TM^ in PD patients with controls. Although only eight people were included in each group, significant improvements in balance, gait speed and mobility were observed after a six-week training program. Increased cortical activity was observed in healthy individuals and in PD patients when they were learning new visuomotor tasks. Cortical activity decreased as learning progressed in healthy individuals, but patients with PD still needed to employ much attention even after several sessions of visuomotor training.^20^

VR dual-task gait training involves executive function,^19^ a predictor of balance deficits in patients with PD.^48^ VR tasks involve gait control and meet environmental demands (e.g. risk detection). Therefore, they can prevent falls and increase functional independence in patients with PD. VR dual-task gait training promotes ecological learning of selecting, planning and monitoring motor programs and of assessing cognitive resources (working memory and attention). These cognitive resources are affected by PD and frequently associated with falls risk and functional dependence. It is important to highlight the increase in motivation promoted by VR, which favours engagement and more efficient motor control and may explain the functional gains even in more severe patients.

Limitations of the present study included the fact that the variability in assessment and training protocols and in VR characteristics hindered meta-analysis. Evaluation follow-up times were also variable and only available in six (Espay et al., 2011;^18^ Mirelman et al., 2011;^19^ Liao et al., 2015;^25^ Ginis et al., 2016;^28^ Strouwen et al., 2017;^30^ Melo et al., 2018^34^) of the 19 studies. New therapeutic strategies for patients with PD, such as VR dual-task gait training, involve integrative and low-cost approaches. Patients with PD must be considered within a biopsychosocial context, as they typically have to deal with the neurodegenerative disease for over 30 years of their life time. VR can be useful for dual-task gait training in patients with PD, providing higher engagement and motivation.

In conclusion, VR dual-task gait training promotes gait improvement in patients with PD. Further studies should evaluate and compare the effects of specific rehabilitation programs in order to provide standardized guidelines for dual-task gait training in PD.
